# Hybrid FFF-CNC Processing of PLA Fixtures with Gyroid Internal Structures

**DOI:** 10.3390/ma19173677

**Published:** 2026-08-29

**Authors:** Emilia Franczyk, Wojciech Zębala

**Affiliations:** Department of Production Engineering and Automation, Faculty of Mechanical Engineering, Cracow University of Technology, 31-155 Cracow, Poland; wojciech.zebala@pk.edu.pl

**Keywords:** hybrid manufacturing, additive–subtractive manufacturing, FFF printing, CNC milling, PLA, gyroid infill, surface roughness, dimensional accuracy

## Abstract

The study presents the results of experimental investigations into hybrid additive–subtractive manufacturing of lightweight PLA fixtures with gyroid internal structures. The proposed manufacturing approach combines FFF (Fused Filament Fabrication) printing with finish CNC milling in order to improve the dimensional accuracy, surface quality, and functional performance of polymer components intended for rapid tooling and functional prototyping applications. Test specimens with gyroid infill densities of 30% and 60% were manufactured from PLA material and subsequently subjected to finish milling under various cutting conditions. Particular attention was paid to the influence of cutting speed and feed per tooth on surface integrity, dimensional stability, and thermal effects occurring during machining. Maintaining the process temperature below the glass transition range of PLA (approximately 50 °C–60 °C) was identified as a critical factor for minimizing deformation and preserving geometric accuracy. The obtained results demonstrate that hybrid FFF-CNC processing enables the fabrication of lightweight yet sufficiently stiff fixtures characterized by improved surface roughness and high dimensional precision. The application of gyroid internal structures significantly reduces component mass while maintaining adequate mechanical rigidity. Moreover, the milled reference surfaces ensure precise positioning and repeatable geometric basing of cooperating machine and robotic assembly elements. The proposed hybrid manufacturing approach shows considerable potential for industrial implementation, particularly in the production of customized positioning fixtures, jigs, and functional tooling components used in automated assembly systems and rapid tooling applications.

## 1. Introduction

In recent years, a dynamic development of additive manufacturing technologies has been observed, with a particular emphasis on methods based on the extrusion of thermoplastic materials, such as FFF. This technology finds widespread application in rapid prototyping, the production of manufacturing fixtures, and the fabrication of functional components with complex geometries. Among the most commonly utilized materials is PLA (*Polylactic Acid*), which, due to its ease of processing, low processing shrinkage, and biodegradability, is widely used in both industrial and research applications [1]. In particular, numerous studies have investigated the influence of filament extrusion rate on the structural, mechanical, and thermal properties of PLA-based 3D-printed components [2]. Liu et al. investigated the effects of key FFF process parameters, including layer thickness, raster angle, extrusion temperature, and printing speed, on the tensile properties and interlayer bonding behavior of PLA components, demonstrating their significant influence on the mechanical performance of 3D-printed parts [3]. Shergill et al. studied the effect of layer thickness on the mechanical properties of additively manufactured PLA specimens, demonstrating that layer thickness has a significant influence on the mechanical performance of the printed components [4]. Budziński et al. analyzed the suitability of PLA and PETG as 3D-printed reference materials for compressive strength testing, analyzing their mechanical performance and the influence of printing parameters on the obtained compressive strength [5].

The modifications of this polymer are extensively analyzed in the literature; Popypovic et al. performed an optimization of PLA printing parameters [6], while other authors investigated the influence of printing speed, nozzle temperature, bed temperature, and layer height on the dimensional accuracy of the parts, indicating the necessity of subsequent post-processing to improve their surface quality [7]. Furthermore, owing to its ability to form strong bonds with synthetic, cellulose, and protein fibers, various fillers can be incorporated into the PLA matrix. This polymer can also be formed into diverse shapes, including films, microcapsules, or hydrogels [8].

Concurrently, the literature indicates that parts fabricated via the FFF method are characterized by limited dimensional and geometric accuracy, as well as relatively high surface roughness, which directly results from the layer-by-layer nature of the manufacturing process [9]. Nevertheless, studies have shown that with proper calibration of the FFF printer, it is possible to obtain components that meet specified dimensional tolerance and fit requirements, confirming the suitability of this technology for critical engineering applications. [10].

Numerous research papers have analyzed the impact of 3D printing parameters -such as layer height, nozzle temperature, printing speed, as well as infill type and density-on the mechanical and geometric properties of PLA prints. Special attention has been paid to modern spatial structures of the TPMS (*Triply Periodic Minimal Surface*) type, including the gyroid structure [9]. These structures are distinguished by a favorable stiffness-to-weight ratio, isotropic stress distribution, and good energy absorption. Research confirms that the use of a gyroid infill enables a significant reduction in the weight of components while maintaining adequate strength properties and controlled porosity. This is particularly important in the context of elements used in biomedicine, robotics, and the automation of production processes [11,12].

Despite the many advantages of FFF technology, the primary limitations remain insufficient surface quality and geometric accuracy of components intended for functional applications [13]. In response to these issues, hybrid technologies combining additive manufacturing with subtractive processing are attracting increasing interest. The literature emphasizes that the application of finish CNC milling significantly reduces surface roughness, while also improving the dimensional accuracy and surface quality of the locating features of FFF-printed components [14]. This is of crucial importance for positioning elements and manufacturing fixtures, where high geometric repeatability and precise locating of mating parts are required. In this context, the extension of the UMRM (*Unified Manufacturing Resource Model*) approach enables the modeling of fixtures, loading devices, workpieces, and the processes themselves. As demonstrated by Vivhare et al., the correct selection of components constitutes a key factor influencing the final accuracy and quality of the product [15].

Authors of many publications draw attention to the difficulties associated with the machining of polymer materials produced additively. Parts fabricated using this method, when subjected to finishing operations via subtractive machining, often exhibit anisotropy of properties as well as dimensional and geometric errors [16]. Kowalska et al. showed that the structure obtained during the 3D printing process influences the mechanical properties and material behavior during subsequent machining [17]. Temperature is a crucial factor during printing; therefore, during the subsequent machining stages, the temperature in the cutting zone must be controlled to prevent component deformation or loss of dimensional stability [18]. In the case of PLA, exceeding the glass transition temperature of the material leads to localized deformations, degradation of surface quality, and a loss of dimensional accuracy [19]. For this reason, the selection of cutting parameters, such as cutting speed and feed per tooth, has a significant impact on the course of the process and the final quality of the product [20,21,22].

An analysis of the available literature indicates that despite the growing interest in hybrid additive-subtractive manufacturing, there is still a lack of comprehensive research regarding the finish milling of PLA components with gyroid structures intended for rapid tooling and functional prototyping applications. In particular, the influence of milling parameters on the geometric accuracy, surface roughness, and thermal stability of lightweight spatial structures remains insufficiently understood. Therefore, it is justified to conduct research on the optimization of the hybrid FFF-CNC process for components utilized as lightweight positioning elements and manufacturing fixtures in robotic systems.

The aim of this study was to evaluate the applicability of hybrid FFF-CNC manufacturing for producing lightweight PLA fixtures with gyroid internal structures. Particular attention was paid to the influence of finish milling parameters on the surface quality, dimensional accuracy, and thermal stability of components intended for rapid tooling and robotic assembly applications.

## 2. Materials and Methods

### 2.1. Geometry and Structure of Test Specimens

The test specimens were manufactured using the FFF process. The geometry of the samples was designed to enable the evaluation of the influence of hybrid additive–subtractive processing on surface quality, dimensional accuracy, and geometric stability of PLA components with internal gyroid structures. The slicer used was Bambu Studio software (Bambu Lab, Shenzhen, China). The main printing parameters were a layer height of 0.2 mm, a line width of 0.42 mm, an infill speed of 250 mm/s, and an outer wall speed of 200 mm/s. The nozzle temperature was set to 220 °C, while the build plate temperature was 60 °C.

**External dimensions of the specimens**

**Figure 1:**
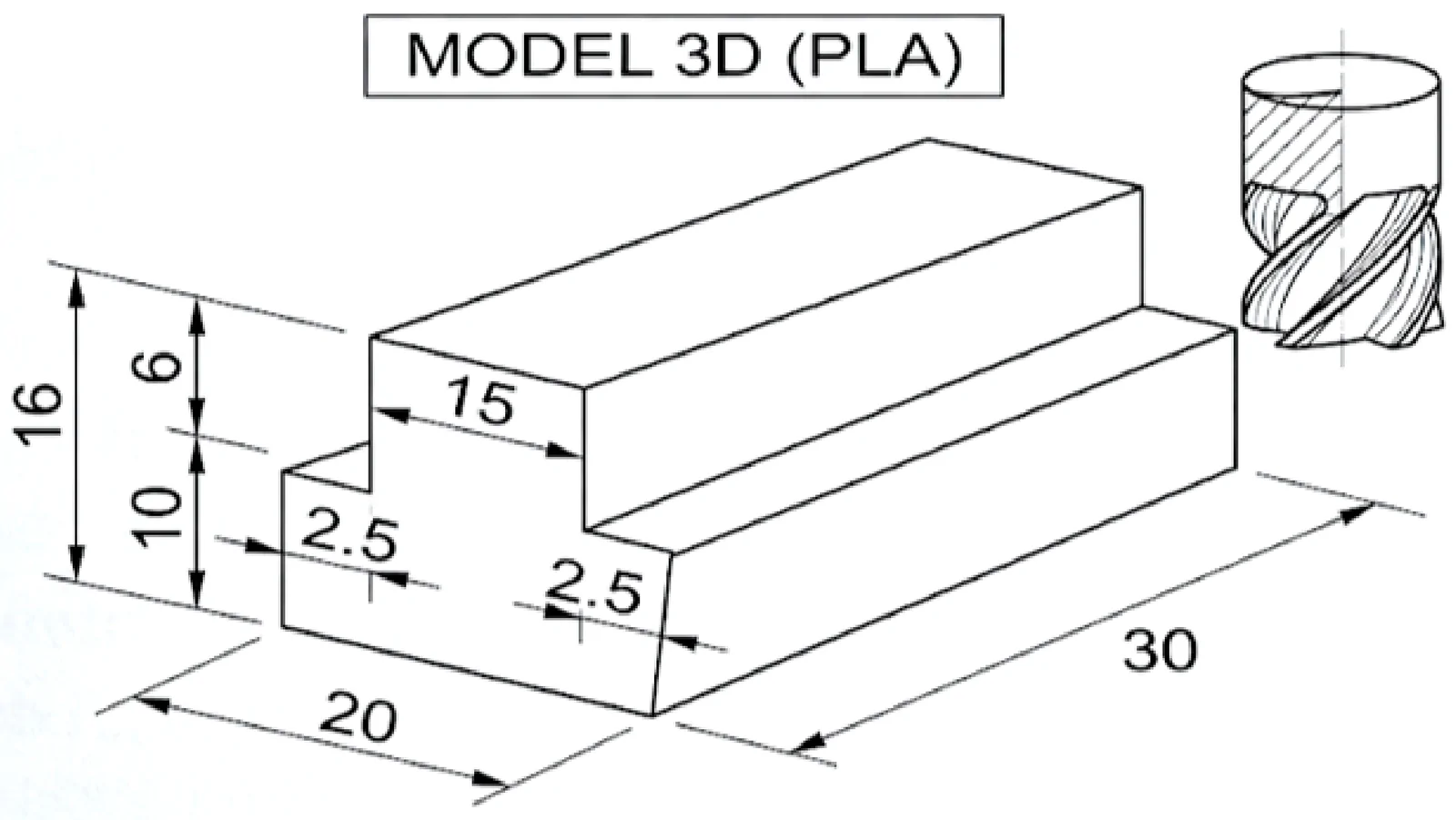
Geometry of the specimen.

The nominal dimensions of the specimens are presented below in Figure 1:

- Overall length: 30 mm;
- Base width: 20 mm;
- Upper step width: 15 mm;
- Side step width: 2 × 2.5 mm (symmetrical);
- Base height: 10 mm;
- Upper step height: 6 mm;
- Total height: 16 mm.

The geometry of the specimens included a stepped upper surface intended for finish milling operations and dimensional accuracy assessment.

**Internal structure and FFF printing parameters**

**Figure 2:**
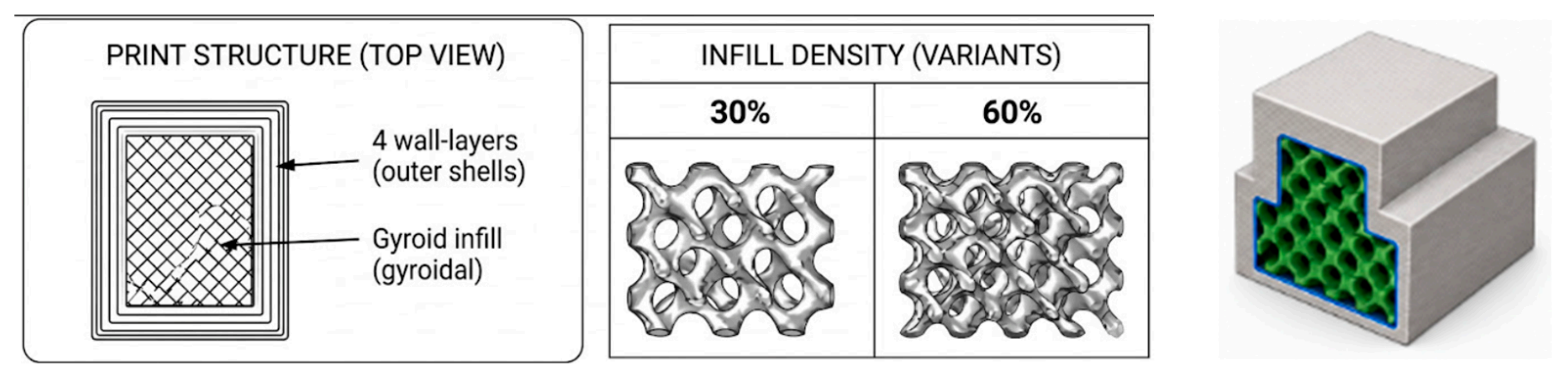
Internal structure and FFF.

The specimens were manufactured from PLA (*Polylactic Acid*) filament using FFF technology. To ensure sufficient rigidity of the lightweight structures, a gyroid-type internal infill was applied, as shown in Figure 2.

The following printing parameters were used:

- Material: PLA;
- Number of outer walls (perimeters): 4;
- Infill type: gyroid;
- Infill density variants: 30% and 60%.

**Figure 3:**
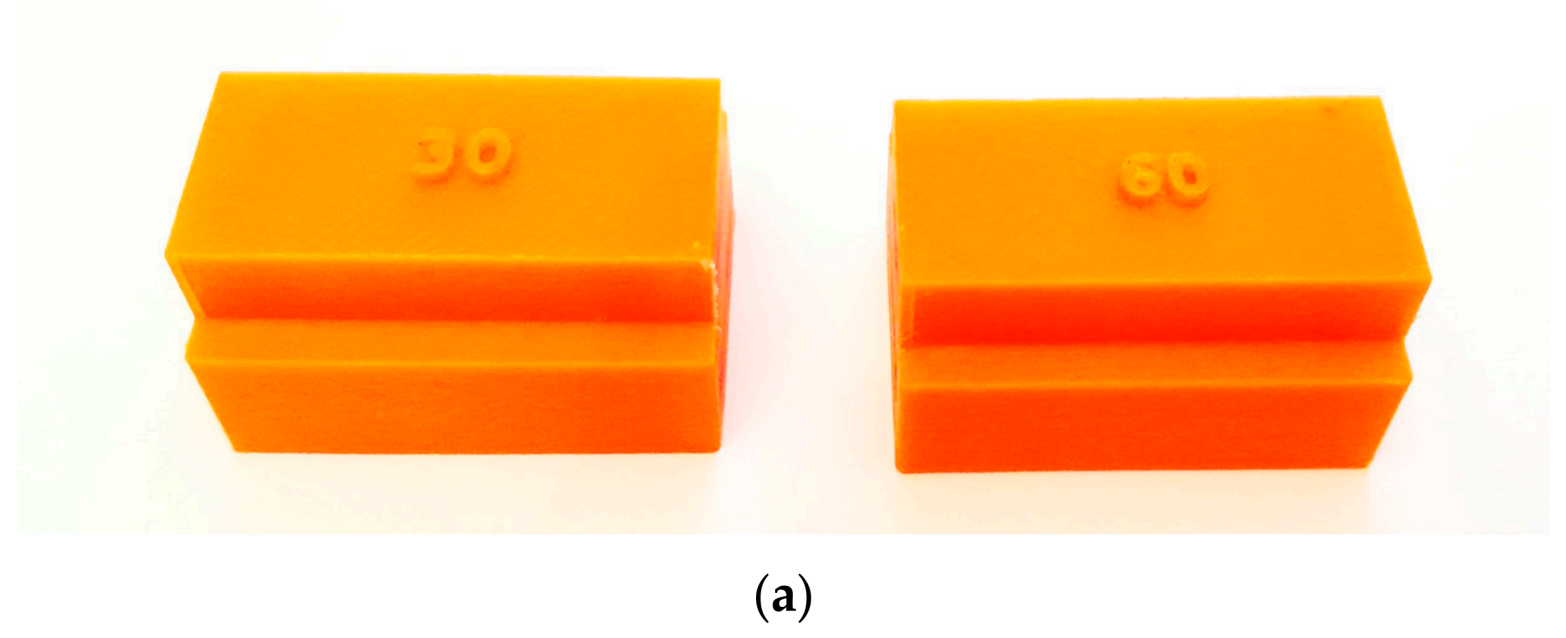

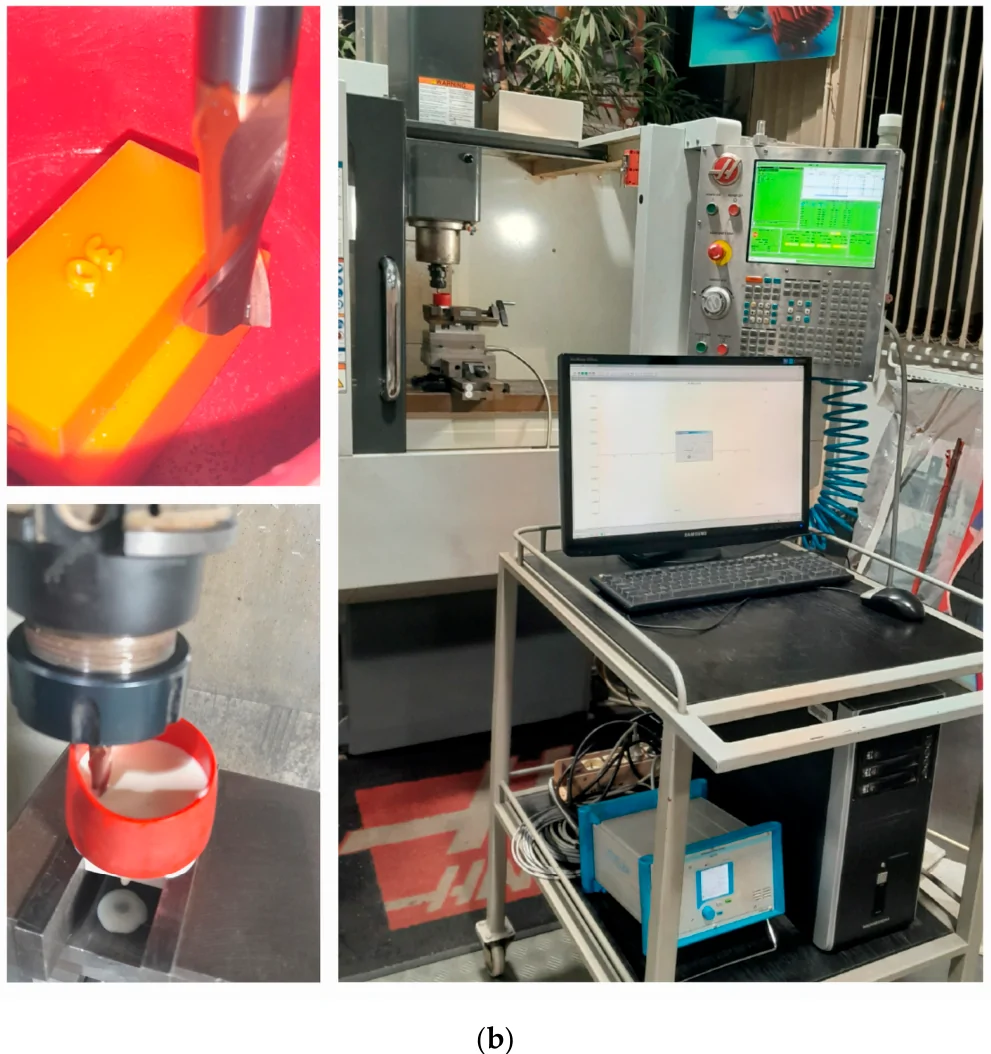
Examples of specimens produced using a 3D printer (**a**), and the research workstation used for milling (**b**).

Figure 3 shows the specimens produced using a 3D printer, as well as the research workstation used for milling.

### 2.2. Machining Conditions

After additive manufacturing, the specimens were subjected to finish milling in order to improve the surface quality and dimensional accuracy of the functional surfaces.

The machining process was performed using climb milling with intensive cooling conditions to minimize thermal effects and prevent local softening of the PLA material.

**Cutting tool**

**Figure 4:**
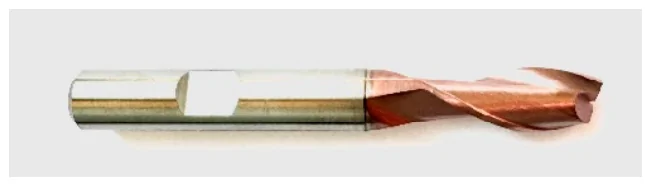
Milling cutter used in the experiments.

The following cutting tools were applied during the experiments, Figure 4:

- Tool type: two-flute end mill;
- Tool diameter (d): Ø8 mm;
- Milling strategy: climb milling.

The milling tool used in the study was an ATORN end mill MPN 10910424 (ATORN, HAHN+KOLB Werkzeuge GmbH, Ludwigsburg, Germany) made of SC material with an ULTRA surface finish, featuring two cutting edges, a 30° helix angle, an 8 mm cutting diameter, a 19 mm cutting edge length, and an 8 mm shank diameter.

The machining parameters were selected to maintain the process temperature below the glass transition temperature of PLA and to reduce the risk of deformation of the lightweight structures (Table 1).

The remaining cutting parameters are presented in Table 2.

**Figure 5:**
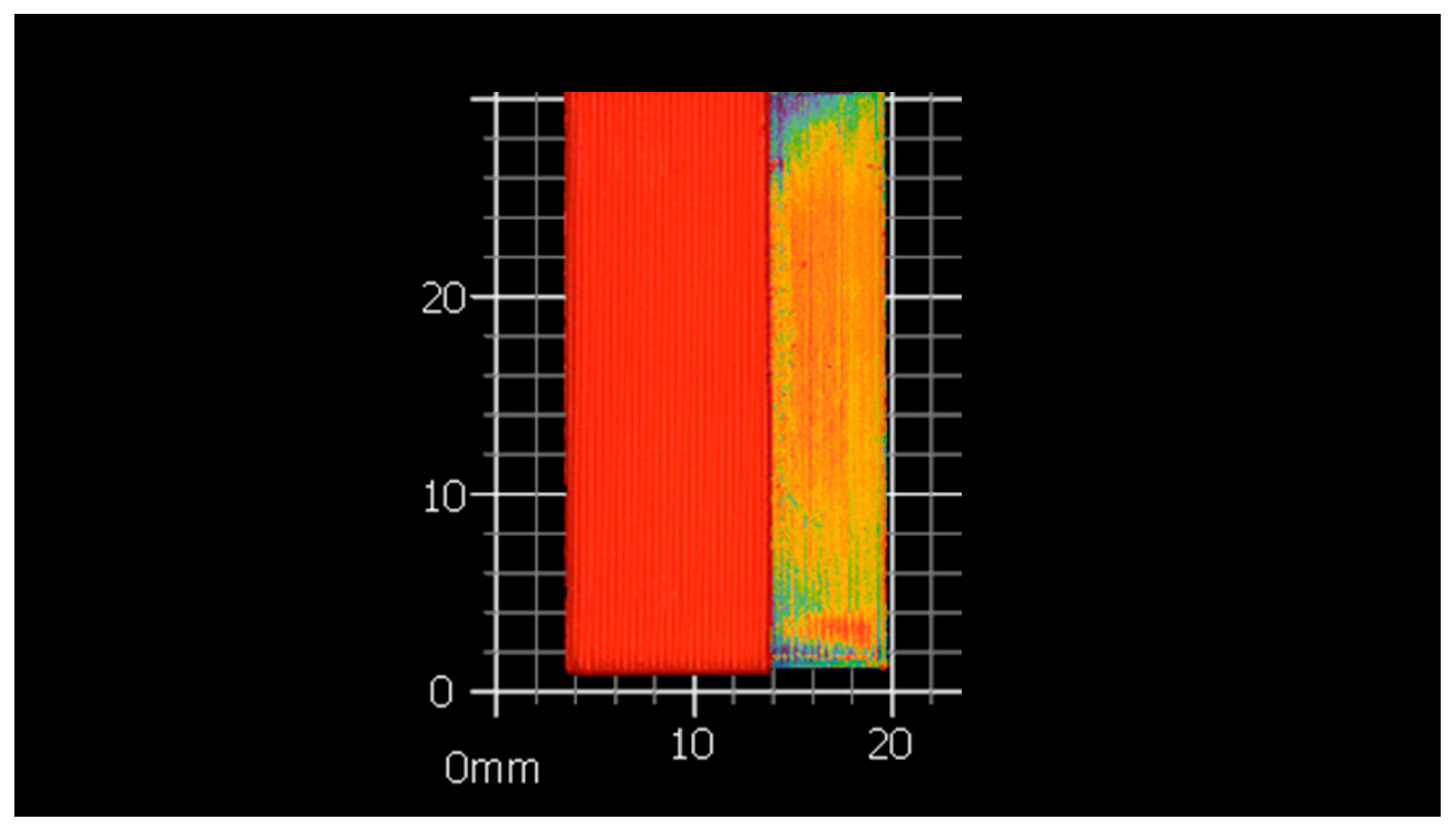
Representative 3D surface topography of the machined workpiece obtained using a non-contact optical measurement system.

The machined surfaces of the workpieces were analyzed using a VR-3200 One-Shot 3D Measuring Microscope, a non-contact optical 3D surface measurement system. Representative measurement results are presented in Figure 5.

The surface quality assessment included the analysis of 3D surface topography and linear surface characteristics, including surface roughness parameters and linearity deviation. These parameters were determined in two mutually perpendicular directions, as summarized in Table 3.

## 3. Results and Discussion

The results of the 3D surface topography measurements are presented in Table 4. Analysis of the obtained images revealed distinct differences in the surface geometric structure, depending on the applied machining parameters, including feed rate, cutting speed, and material infill density. The observed variations indicate that these factors have a significant effect on the quality of the machined surfaces.

**Figure 6:**
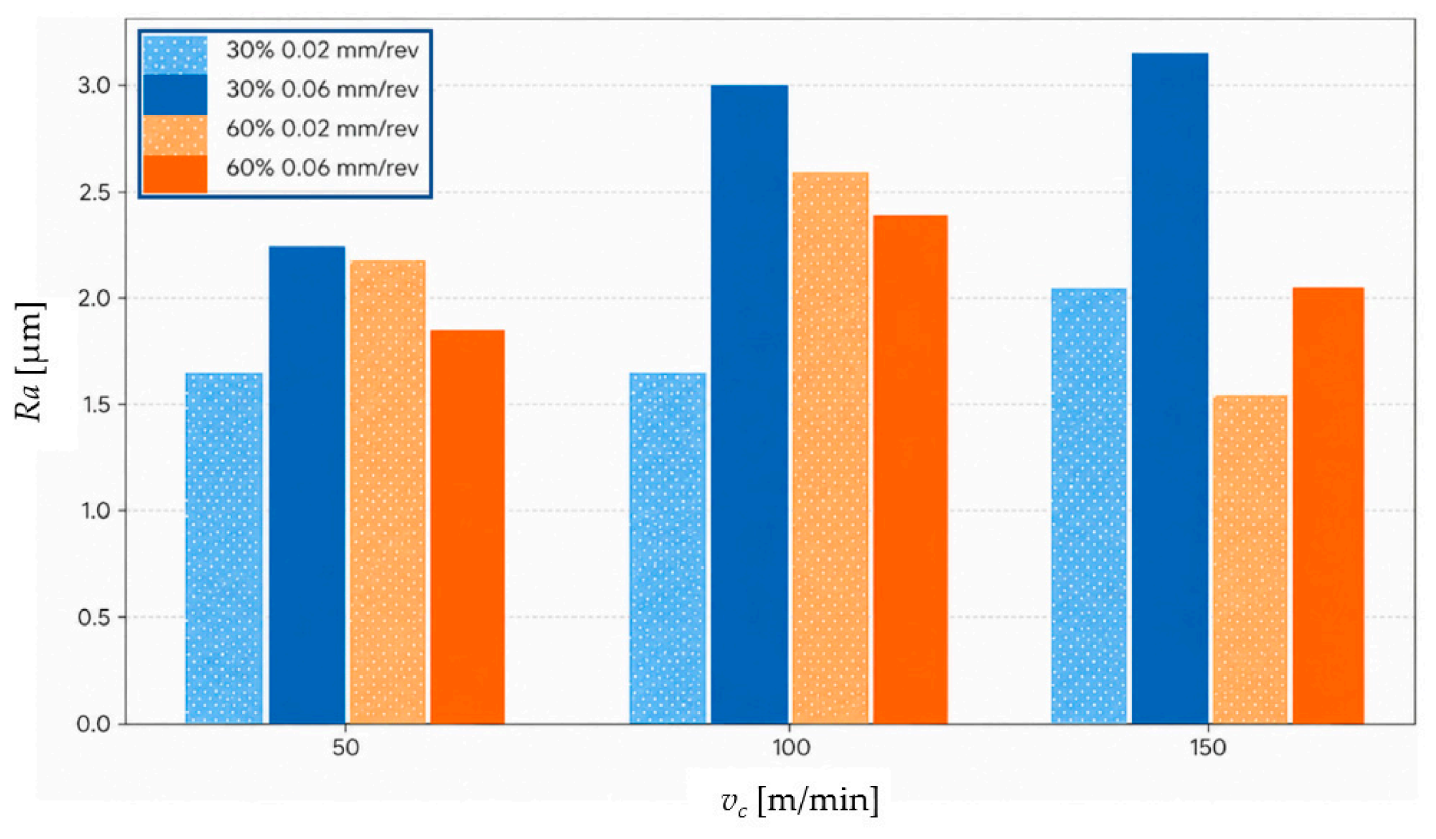
Surface roughness parameter *Ra* as a function of feed rate, cutting speed, and infill density, measured along the milling path.

**Figure 7:**
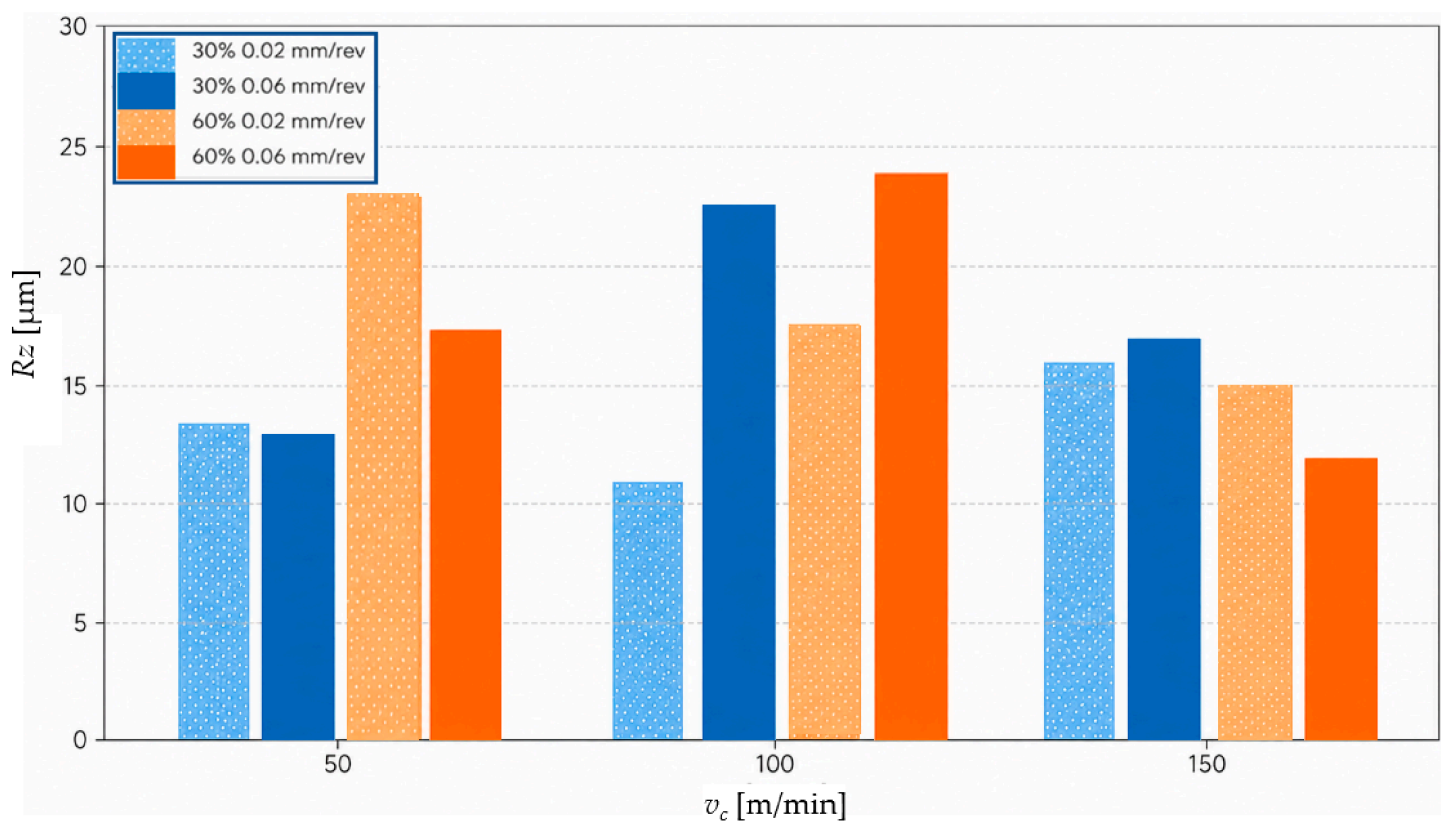
Surface roughness parameter *Rz* as a function of feed rate, cutting speed, and infill density, measured along the milling path.

Figure 6 and Figure 7 present the results of the surface roughness measurements for the *Ra* and *Rz* parameters obtained at different cutting speeds and feed rates. The roughness measurements were performed along the milling path, enabling an assessment of the influence of the cutting parameters on the quality of the machined surface.

The analysis of the results showed that, for most of the investigated conditions, increasing the feed per tooth resulted in a reduction in the *Ra* surface roughness parameter. In contrast, for specimens with a 30% infill density, an increase in cutting speed led to higher *Ra* values. For specimens with a 60% infill density, the relationship was non-linear: the *Ra* value initially increased with increasing cutting speed and subsequently decreased.

**Figure 8:**
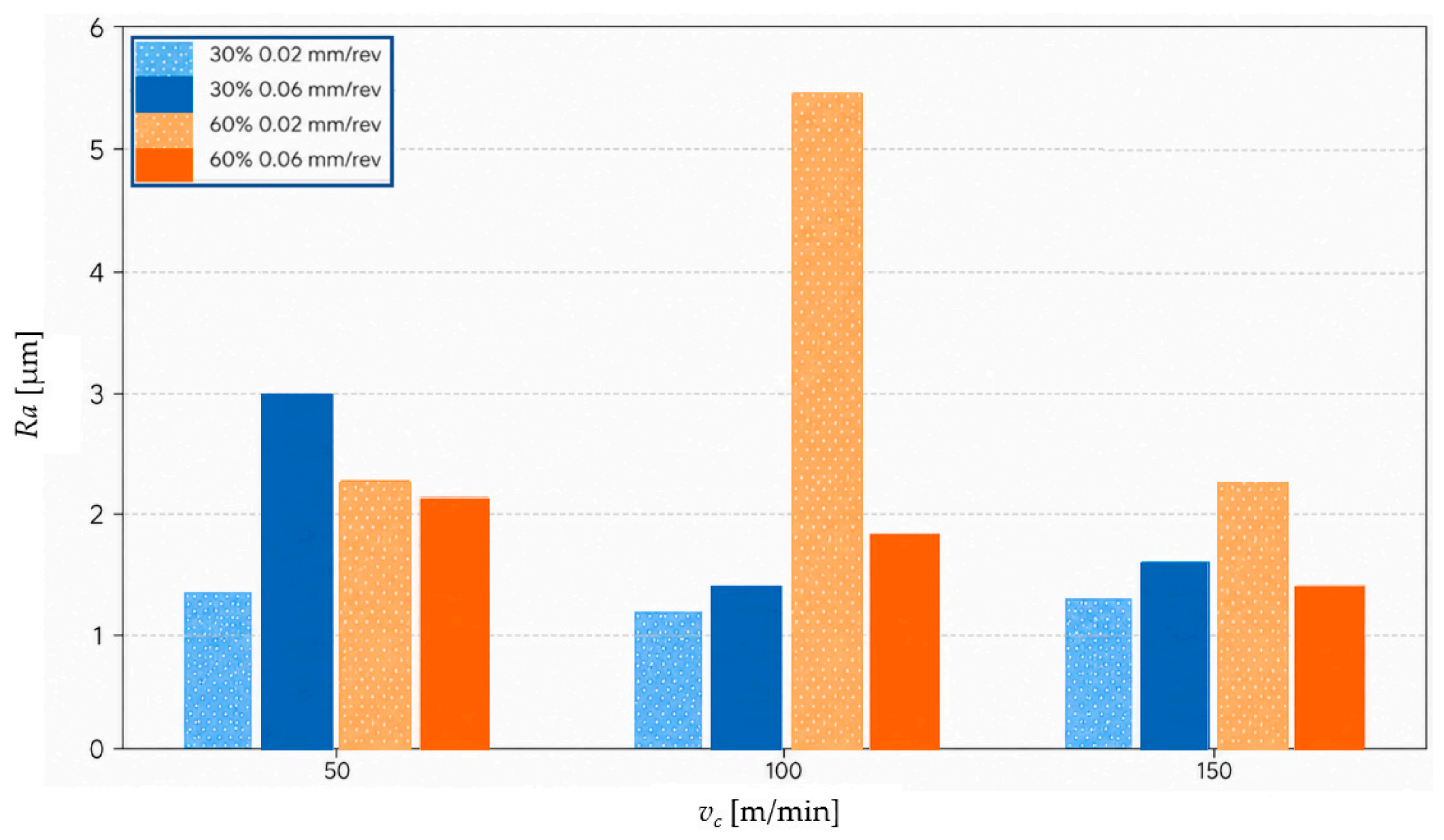
Surface roughness parameter *Ra* as a function of feed rate, cutting speed, and infill density, measured perpendicular to the milling path.

**Figure 9:**
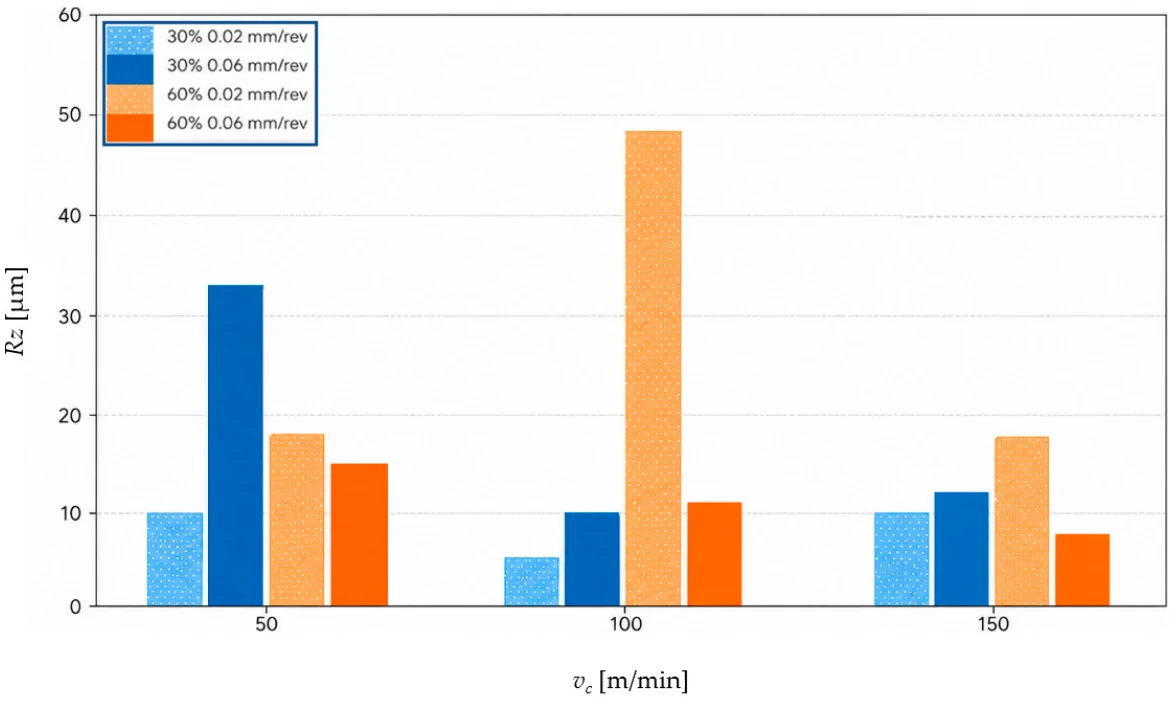
Surface roughness parameter *Rz* as a function of feed rate, cutting speed, and infill density, measured perpendicular to the milling path.

Figure 8 and Figure 9 present the results of the *Ra* and *Rz* surface roughness parameters as a function of cutting speed, feed rate, and infill density. The surface roughness measurements were performed perpendicular to the milling path, allowing the influence of the investigated machining parameters on the surface quality across the machining marks to be evaluated. The results showed that, at the lower feed rate, both infill density and cutting speed had a significant effect on the surface roughness values. In contrast, at the higher feed rate, only minor differences were observed between the tested conditions, despite variations in cutting speed and infill density.

For the *Ra* parameter, the lowest value measured along the milling path was obtained with a 60% 3D printing infill, a cutting speed of 150 m/min, and a feed rate of 0.02 mm/rev. In contrast, for the perpendicular to the milling path direction, the lowest *Ra* value was achieved with a 30% infill, a cutting speed of 150 m/min, and a feed rate of 0.02 mm/rev.

Regarding the *Rz* parameter, the lowest values for both the along the milling path and perpendicular to the milling path measurement directions were obtained with a 30% 3D printing infill, a cutting speed of 150 m/min, and a feed rate of 0.02 mm/rev.

### 3.1. Effect of Cutting Parameters on Surface Roughness

Table 5 summarizes the results of the surface roughness measurements, including the *Ra* and *Rz* parameters determined along and perpendicular to the tool feed direction, together with the linearity deviation values obtained for the twelve investigated combinations of machining parameters.

#### 3.1.1. Effect of Feed per Tooth

The analysis of the results revealed a clear and consistent relationship between the feed per tooth (*f_z_*) and the resulting surface roughness measured both along and perpendicular to the tool feed direction. In contrast to conventional metal machining theory, where an increase in feed per tooth geometrically leads to higher surface roughness, the milling of FFF-manufactured thermoplastic polymers exhibited the opposite trend. Increasing the feed per tooth from 0.02 mm/tooth to 0.06 mm/tooth resulted in a reduction in the *Ra* surface roughness parameter for the vast majority of the investigated machining conditions.

This behavior can be explained by the Minimum Chip Thickness (MCT) effect. At a feed per tooth of 0.02 mm/tooth, the undeformed chip thickness approaches the cutting-edge radius, causing the material removal process to be dominated by plowing rather than by effective shearing. As a consequence, the thermoplastic material undergoes elastic deformation beneath the cutting edge and partially recovers after tool passage, leading to the detachment of microscopic fragments from the filament structure, burr formation, and increased *Ra* and *Rz* values. This phenomenon is particularly evident for measurements performed perpendicular to the tool feed direction. For example, the *Ra* value obtained for Workpiece 1 was 3.10 μm, whereas for Workpiece 2, machined at the same cutting speed (*v_c_* = 50 m/min) but with a higher feed per tooth (*f_z_* = 0.06 mm/tooth), the *Ra* value decreased to 1.36 μm, corresponding to a reduction in more than 56%.

As the feed per tooth increased to 0.06 mm/tooth, the undeformed chip thickness exceeded the critical minimum chip thickness, allowing the cutting edge to engage in a stable shearing process instead of material plowing. This transition resulted in more regular chip formation and improved surface generation, producing smoother tool marks. Thermal effects also contributed significantly to the observed behavior. At lower feed rates, the cutting tool performs a greater number of revolutions per unit length of the workpiece, promoting heat accumulation within the cutting zone. Thermoplastic polymers such as PLA are characterized by low thermal conductivity and a relatively low glass transition temperature; therefore, localized heat accumulation promotes material softening and smearing along the boundaries of adjacent deposited filaments, leading to increased surface roughness. At the higher feed per tooth, a larger fraction of the generated heat is removed with the chip, while the tool-workpiece contact time is reduced, thereby limiting the thermal degradation of the machined polymer and improving the surface finish.

#### 3.1.2. Effect of Cutting Speed

The effect of cutting speed (*v_c_*) on surface roughness was less pronounced than that of feed per tooth and exhibited a strong interaction with the remaining machining parameters. For workpieces with an infill density of 30% machined at the lower feed per tooth (*f_z_* = 0.02 mm/tooth-tests 1, 3, and 5), increasing the cutting speed from 50 to 150 m/min resulted in a gradual increase in the *Ra* value measured along the tool feed direction (2.19, 2.92, and 3.02 μm, respectively). This trend may be attributed to enhanced thermal effects occurring at higher cutting speeds. In contrast, at the higher feed per tooth (*f_z_* = 0.06 mm/tooth), the *Ra* values measured along the tool feed direction did not exhibit a monotonic dependence on cutting speed, indicating that, within the investigated range, the influence of cutting speed was secondary to that of feed per tooth.

The lowest *Ra* values measured along the tool feed direction were obtained for the combination of *v_c_* = 100 m/min and *f_z_* = 0.06 mm/tooth with a 30% infill density (Test 4, *Ra* = 1.56 μm), as well as for *v_c_* = 150 m/min and *f_z_* = 0.02 mm/tooth with a 60% infill density (*Ra* = 1.50 μm, Test 11). These findings indicate the existence of an optimum combination of cutting parameters, which depends on the thermomechanical properties of the machined polymer.

### 3.2. Effect of Infill Density on Surface Quality

#### 3.2.1. Surface Roughness Measured Perpendicular to the Tool Feed Direction

A comparative analysis of the results obtained for workpieces with 30% and 60% infill densities revealed a seemingly counterintuitive relationship: increasing the infill density did not improve the quality of the machined surface but, in many cases, resulted in higher surface roughness measured perpendicular to the tool feed direction. This effect was particularly pronounced for test 9 (*v_c_* = 100 m/min, *f_z_* = 0.02 mm/tooth, 60% infill), where the *Ra* and *Rz* values reached 5.60 μm and 51.0 μm, respectively. These values were several times higher than those obtained under the corresponding machining conditions for the 30% infill workpiece (*Ra* = 1.50 μm and *Rz* = 10.4 μm + test 3).

This behavior can be attributed to the non-uniform dynamic stiffness of the internal structure. Unlike conventionally manufactured components, FFF-produced workpieces are not homogeneous solids but consist of a network of interconnected deposited filaments. At an infill density of 60%, the internal rib structure is considerably denser than at 30%, causing the cutting tool to periodically encounter regions of substantially different local stiffness during transverse machining. Highly rigid intersections of the filament network alternate with less supported regions, resulting in cyclic variations in the cutting force. These variations induce tool micro-vibrations, which are manifested as periodic surface irregularities when roughness is measured perpendicular to the filament orientation. Similar vibration-related effects resulting from variations in structural stiffness during milling of additively manufactured components have been reported in the literature [23].

An additional factor contributing to the deterioration of the surface quality at higher infill densities is the reduced efficiency of heat dissipation from the cutting zone. Thermoplastic polymers exhibit inherently low thermal conductivity, and increasing the infill density from 30% to 60% reduces the volume of internal air cavities within the workpiece. At lower infill densities, these cavities facilitate heat transfer within the internal structure and promote convective heat dissipation from the cutting zone. In contrast, the denser internal structure at 60% infill promotes greater heat accumulation in the tool–workpiece contact region. The resulting local temperature rise leads to softening and smearing of the deposited filament walls adjacent to the machined surface, particularly when the roughness is evaluated perpendicular to the filament orientation, thereby increasing the measured *Ra* and *Rz* values.

#### 3.2.2. Linearity Deviation

The results of the linearity deviation measurements indicate consistently higher values for workpieces with a 60% infill density compared to those with a 30% infill density. The average linearity deviation for workpieces 7–12 (60% infill) was 0.039 mm, whereas for workpieces 1–6 (30% infill) it was 0.029 mm, representing an increase of approximately 34%.

This behavior can be explained by the residual stresses generated during the Fused Filament Fabrication process. During printing, each deposited polymer layer undergoes thermal contraction as it cools, leading to the accumulation of tensile residual stresses within the plane along the build platform. As the infill density increases, the volume of material subjected to thermal shrinkage also increases, resulting in higher levels of residual internal stresses. Milling removes the outer material layer, thereby disturbing the internal stress equilibrium of the workpiece. The subsequent stress relaxation within the denser infill structure causes local deformation (warping) of the workpiece, which is manifested as an increased linearity deviation. A similar stress-relief mechanism has been reported for machined components containing residual thermal or welding stresses [24].

Furthermore, the higher cutting forces associated with the greater volume of material removed from workpieces with a denser internal structure may induce elastic deflections of the entire machining system, including the machine tool, fixture, cutting tool, and workpiece. These deflections adversely affect the geometric accuracy of the machined surface and contribute to the observed increase in linearity deviation.

#### 3.2.3. Analysis of Variance (ANOVA)

Data analysis was performed using analysis of variance (ANOVA). The results of the ANOVA are presented in Table 6, where DF denotes the degrees of freedom, Adj SS represents the adjusted sum of squares, and Adj MS represents the adjusted mean square.

Statistical analysis showed that feed rate was the factor with the strongest and highly statistically significant effect on the surface roughness parameter *Ra*. Changes in feed rate resulted in pronounced variations in surface roughness, with its effect being considerably greater than that of the other analyzed factors. The infill percentage also had a statistically significant effect on *Ra*, indicating that changes in the infill percentage affect the resulting surface quality, although this effect was weaker than that of feed rate. In contrast, cutting speed did not have a statistically significant effect on *Ra*. A statistically significant interaction between cutting speed and feed rate indicates that the effect of one parameter on surface roughness depends on the level of the other; therefore, these parameters should be considered jointly when optimizing the machining process. No statistically significant interactions were observed between cutting speed and infill percentage or between feed rate and infill percentage, indicating that these factors affect surface roughness largely independently of each other. The analysis also revealed a statistically significant three-way interaction between cutting speed, feed rate, and infill percentage, suggesting that the simultaneous variation in all three process parameters affects *Ra* in a more complex manner than would be expected from the individual factors or their two-way interactions. In practice, this highlights the need to consider the combined selection of all investigated technological parameters when optimizing the machining process.

## 4. Potential Applications of Hybrid-Manufactured PLA Parts: A Case Study

Positioning components play a crucial role in ensuring the accurate positioning, orientation, and repeatability of operations performed by industrial robots with respect to predefined reference datums. They are responsible for maintaining the geometric stability of the system and ensuring repeatable assembly conditions during robot operation, component replacement, and maintenance procedures.

The primary functions of positioning components include:

- Precise definition of the robot arm reference position;
- Centering and guiding of mating components;
- Ensuring repeatability of the robot motion trajectory;
- Accurate positioning of tools and robotic end-effectors;
- Minimizing assembly errors and compensating for mechanical clearances.

Typical positioning components include:

- Base plates with locating holes;
- Centering bushings;
- Positioning brackets;
- Locating pins;
- Slotted guide rails;
- Mounting adapters for robotic tools and end-effectors.

The combination of FFF technology and finish CNC milling enables the manufacture of lightweight components with complex geometries while ensuring high-quality functional surfaces. Finish milling significantly improves:

- Dimensional and geometrical accuracy;
- Surface roughness;
- Hole coaxiality and positional accuracy;
- The quality of datum and mating surfaces.

An additional advantage of the proposed approach is the possibility of employing gyroid internal structures, which significantly reduce the component weight while maintaining sufficient structural stiffness. In robotic applications, this translates into lower inertial forces and improved dynamic performance of the system.

**Figure 10:**
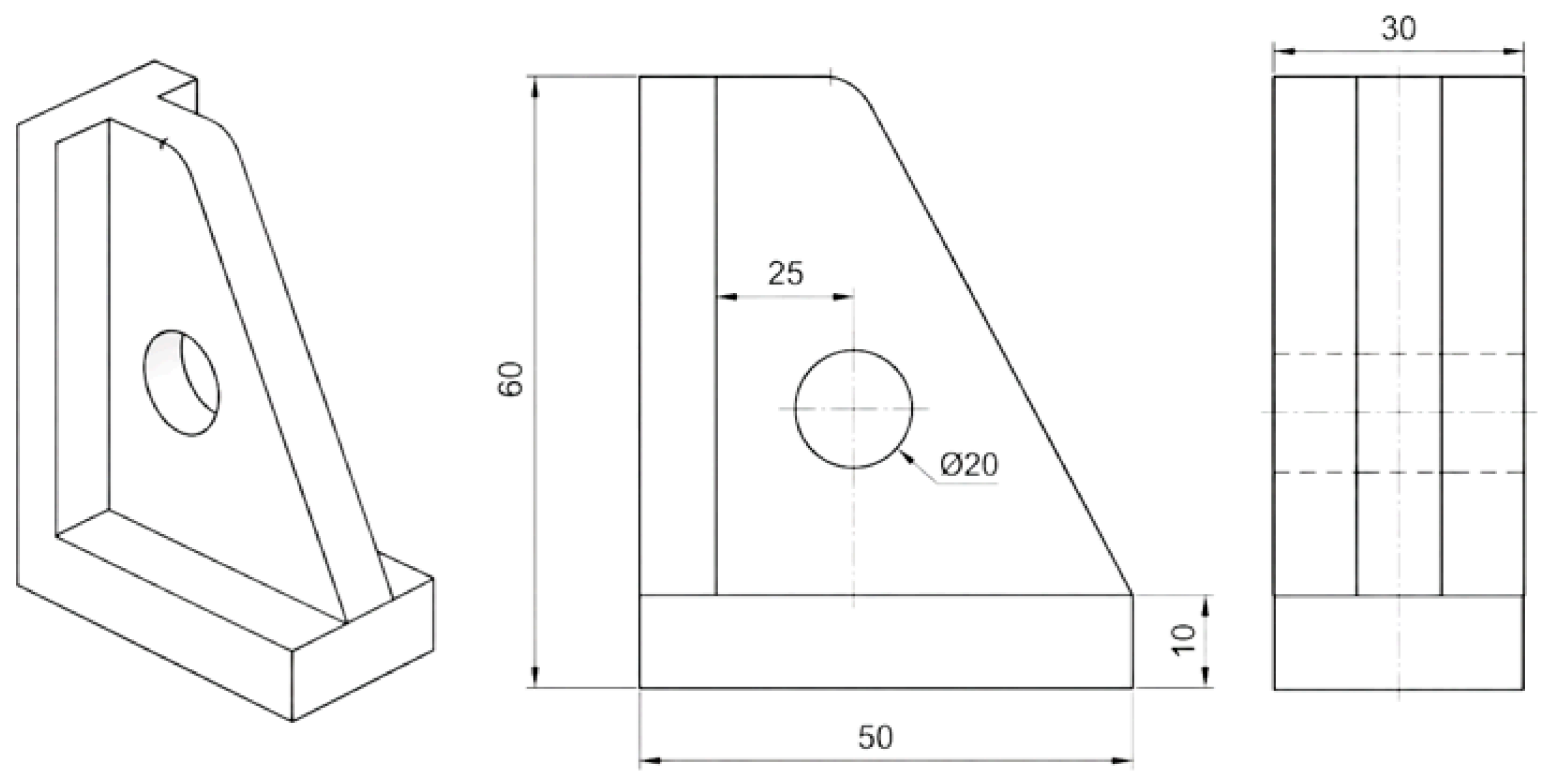
CAD model of the bracket.

To validate the findings presented in the previous section, a representative positioning component used in robotic assembly systems—a positioning bracket shown in Figure 10—was selected as a case study. The component was manufactured using the FFF process and subsequently subjected to finish CNC milling. The machining was performed using the optimum process parameters identified in the experimental study: a cutting speed of *v_c_* = 100 m/min, a feed per tooth of *f_z_* = 0.06 mm/tooth, and an infill density of 30% (test 4). After printing, the average surface roughness was measured as *Ra* = 12.87 µmm and average *Rz* value was 57.01 µm.

The printed bracket was subjected to milling of the mounting surface. The first machining trial was carried out using a cutting depth of *a_p_* = 6 mm, which had been previously determined based on earlier investigations. The second trial was performed with an increased cutting depth of *a_p_* = 15 mm.

Table 7 presents the surface profiles obtained after machining, based on which the surface roughness parameters *Ra* and *Rz* were determined. The table also reports the corresponding values of these parameters, expressed as the arithmetic mean of three measurements.

The obtained results demonstrate that the hybrid manufacturing process enables a substantial improvement in surface quality. The application of milling reduced the surface roughness parameter *Ra* by approximately 94.5%, from 13.55 µm to 0.74 µm (example shown in Table 6). Furthermore, optimization of the cutting parameters, particularly the increase in the axial depth of cut, resulted in an additional reduction in *Ra* by approximately 13.5%, reaching a final value of 0.64 µm. A similar trend was observed for the *Rz* parameter. Milling reduced *Rz* by approximately 91.4%, from 58.68 µm to 5.05 µm. Increasing the axial depth of cut further decreased *Rz* by approximately 12.3%, resulting in a final value of 4.43 µm. For the analyzed component, the average surface roughness calculated from three values before milling was *Ra* = 12.87 µm and *Rz* = 57.01 µm. After milling with an axial depth of cut of a_p_ = 6 mm, the average values decreased to *Ra* = 0.79 µm and *Rz* = 4.40 µm, corresponding to reductions in approximately 93.9% and 92.3%, respectively. These findings confirm that appropriate selection of machining parameters can further enhance the surface finish achieved by hybrid manufacturing.

## 5. Conclusions

Based on the experimental investigations conducted in this study and the analysis of the obtained results, the following conclusions can be drawn regarding the influence of milling parameters and infill density on the surface quality of FFF-manufactured PLA components:

The feed per tooth *f_z_* was identified as the dominant machining parameter affecting the surface quality of milled PLA/FFF specimens. Increasing the feed per tooth from 0.02 to 0.06 mm/tooth consistently reduced the surface roughness (*Ra*) for the majority of the investigated machining conditions. This phenomenon can be attributed to the transition of the cutting process from a ploughing-dominated regime to effective shearing, i.e., exceeding the minimum uncut chip thickness characteristic of the investigated polymer and cutting tool geometry.

The influence of the cutting speed (*v_c_*) on surface roughness was less pronounced and strongly dependent on its interaction with the remaining process parameters. Within the investigated range (50–150 m/min), no monotonic relationship between cutting speed and surface roughness was observed, indicating the existence of an optimum cutting speed determined by the feed per tooth and the infill density.

A higher infill density (60% compared with 30%) unexpectedly deteriorated the machined surface quality in the direction transverse to the feed and increased the flatness deviation. This effect may be associated with the non-uniform mechanical response of the internal cellular structure, which should be experimentally verified in future studies.

The most favorable surface quality was achieved for the following combination of machining parameters: cutting speed (*v_c_* = 100) m/min, feed per tooth (*f_z_* = 0.06) mm/tooth, and an infill density of 30% (Test No. 4). Under these conditions, the lowest surface roughness values were obtained, namely *Ra* = 1.56 μm measured along to the feed direction and *Ra* = 1.30 μm measured perpendicular to the feed direction.

The analysis of the surface morphology of the manufactured bracket demonstrates that the quality of FFF-produced surfaces can be significantly enhanced through the application of a hybrid manufacturing approach combining additive manufacturing and CNC milling. Furthermore, optimization of the machining parameters leads to additional improvements in surface finish, enabling the production of functional surfaces that meet the required quality without the need for subsequent finishing operations.

In future work, predictive models will be developed for the optimization of both FFF and CNC milling parameters to support the industrial implementation of the proposed hybrid manufacturing process. In addition, comprehensive investigations of the mechanical properties of hybrid-manufactured components are planned to evaluate the effect of the combined additive–subtractive process on their structural performance and functional applicability.

## Figures and Tables

**Table 1:** Experimental plan for finish milling parameters using an Ø8 mm milling cutter.

No.	*v_c_* [m/min]	*f_z_* [mm/tooth]	Infill Density [%]
1	50	0.02	30
2	50	0.06	30
3	100	0.02	30
4	100	0.06	30
5	150	0.02	30
6	150	0.06	30
7	50	0.02	60
8	50	0.06	60
9	100	0.02	60
10	100	0.06	60
11	150	0.02	60
12	150	0.06	60

**Table 2:** Cutting parameters used in the finish milling experiments.

Depth of cut ap: 6 mm	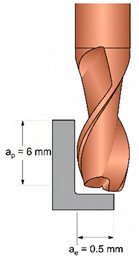
Width of cut ae: 0.5 mm	
Coolant: flood emulsion 15% Blaser coolant	

**Table 3:** Measurement directions used for the evaluation of 3D surface topography, surface roughness parameters, and linearity deviation.

Measured along the milling path.	Measured perpendicular to the milling path
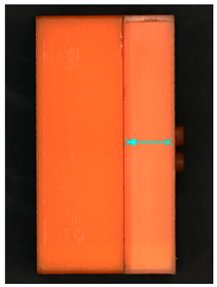	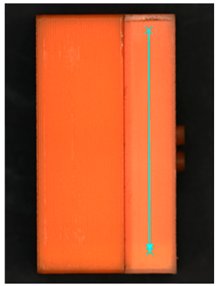

**Table 4:** Surface topography of the obtained samples.

1		2		3	
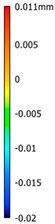	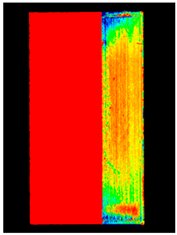	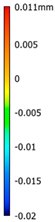	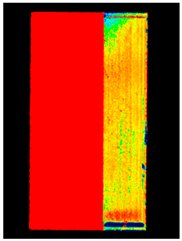	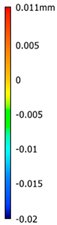	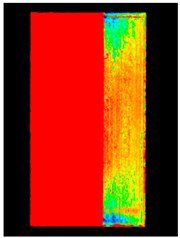
4		5		6	
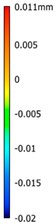	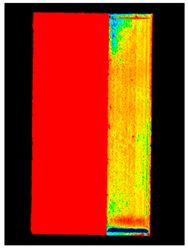	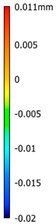	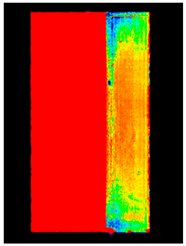	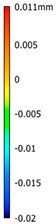	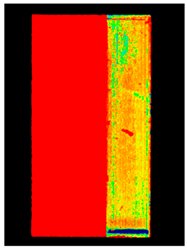
7		8		9	
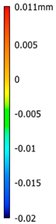	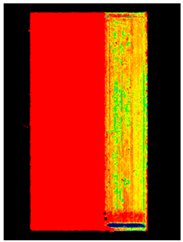	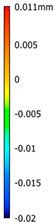	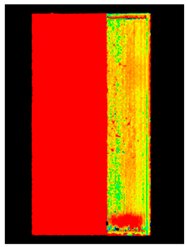	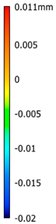	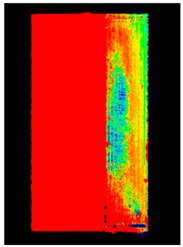
10		11		12	
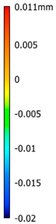	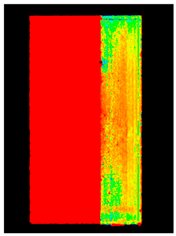	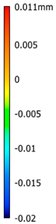	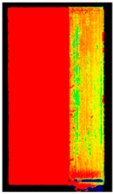	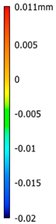	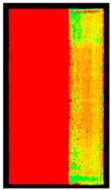

**Table 5:** Surface roughness and linearity deviation measurement results after milling of PLA specimens manufactured by FFF.

No.	*vc* [m/min]	*fz* [mm/tooth]	Infill Density [%]	*Ra*[μm] Along	*Rz*[μm] Along	*Ra* [μm] Perpendicular	*Rz* [μm] Perpendicular	Linearity Deviation [mm]
1	50	0.02	30	2.19	13.00	3.10	34.0	0.033
2	50	0.06	30	1.62	13.48	1.36	11.0	0.033
3	100	0.02	30	2.92	22.93	1.50	10.4	0.031
4	100	0.06	30	1.56	10.76	1.30	6.6	0.034
5	150	0.02	30	3.02	16.91	1.85	14.0	0.028
6	150	0.06	30	1.99	15.80	1.56	13.3	0.017
7	50	0.02	60	2.06	17.77	2.28	19.3	0.043
8	50	0.06	60	1.84	23.30	2.20	15.7	0.030
9	100	0.02	60	2.53	24.00	5.60	51.0	0.044
10	100	0.06	60	2.33	17.45	1.88	11.2	0.039
11	150	0.02	60	1.50	11.87	2.37	17.7	0.045
12	150	0.06	60	1.95	15.00	1.60	9.0	0.030

**Table 6:** ANOVA results for surface roughness (*Ra*). Statistically significant regression coefficients are highlighted in bold.

	DF	Adj SS	F-Value	*p*-Value
***v_c_***	2	0.0231	2.748	0.0842
*f_z_*	1	0.4444	105.680	<0.0001
**Infill density**	1	0.0187	4.441	0.0457
***v_c_*** · ***f_z_***	2	0.0761	9.044	0.00118
***v_c_*** · **Infill density**	2	0.0049	0.579	0.568
***f_z_*** · **Infill density**	1	0.0011	0.264	0.612
***v_c_*** · ***f_z_*** · **Infill density**	2	0.0373	4.431	0.0230
**Error**	24	0.1009		

**Table 7:** Surface profiles of the milled mounting surface together with the corresponding average surface roughness parameters (*Ra* and *Rz*) obtained for axial depths of cut *a_p_* = 6 mm and *a_p_* = 15 mm.

Before Milling		After Milling a_p_ = 6 mm		After Milling a_p_ = 15 mm	
Mean *Ra* 13.55	Mean *Rz* 58.68	Mean *Ra* 0.74	Mean *Rz* 5.05	Mean *Ra* 0.64	Mean *Rz* 4.43
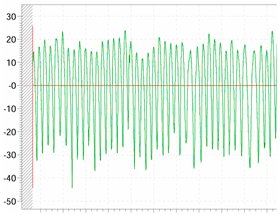		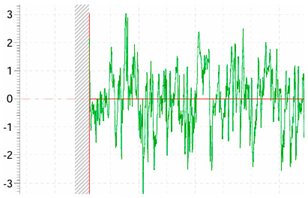		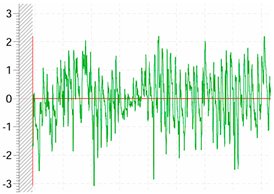	

## Data Availability

The original contributions presented in this study are included in the article. Further inquiries can be directed to the corresponding author.
